# Approach to reflective practice: an epistemological redignification of the professional nurse

**DOI:** 10.1590/1518-8345.0000.3098

**Published:** 2018-11-14

**Authors:** José Luis Medina

**Affiliations:** 1 Universitat de Barcelona, Facultad de Educación, Barcelona, Spain.

**Figure:**
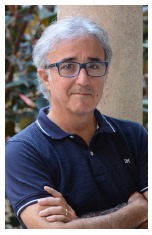

Nowadays, the rapid changes taking place in higher education from proposals issued by the TUNNING project and the European higher education area (EHEA), as well as the radical transformation of its structure, assume a profound change that has a special impact in the teaching-learning processes of nursing. The emergence of a new competency-based curriculum structure, the proposal of new teaching methods explicitly centered on student learning, and the new conception of emerging teaching work in the face of these changes are generating pedagogical-didactic requirements that have no paradigms in the recent history of universities. One of the most outstanding demands a student-centered training that adequately prepares him/her for the complex tasks that nowadays require professional practices of nursing.

One of the central elements of this change has been the profound reconceptualization that has been carried out on the epistemological foundations of actions that professional nurses perform in the exercise of their functions^1^. This re-signification of the nature of knowledge that effectively put professional nurses into play has, in turn, undergone a change in the way they represent, formalize, and transmit them^2^. Two reasons have motivated this change.

First, to paraphrase Schön^3^, we could call it the crisis of expertise or professional competence. This crisis has generated a movement in which the professional skill required for a competent practice of care has ceased to be considered an applied science based on technical or instrumental rationality to rely more and more on a practical-reflexive rationality, which has assumed a redignification of the professional nurse as an epistemic agent^4^.

The first one understands that the good professional praxis of care consists of the application of techniques and procedures, standardized and scientifically validated, for the solution of well-structured and formalized problems. The connection between problems and techniques (means) is learned to be established in university formation through training in the systematic application of scientifically established theories^3^. The processes of identification/ diagnosis of problems and the procedures to solve them are not considered problematic once established, in addition to the difficulties that the student can find to learn them. The second, on the contrary, argues that in professional nursing practice the problems (and their solutions) do not usually present in a structured way. In fact, there is abundant empirical evidence that allows us to affirm that initially they are not usually presented as problems (a situation where at least one known technical solution is available), as well as ambiguous, unclear, and disorganized situations: what Schön^5^ calls a problem situation. In other words, a situation chronologically and cognitively prior to the establishment of the problem. The professional practice of care is saturated with ambiguity and uncertainty and, therefore, requires sufficiently flexible and dynamic knowledge to be able to adapt to the situations of change that constitute it and the ethical problems that are inherent to it. The knowledge that goes far beyond the formal, abstract, and decontextualized theories that are exclusively used by the novices (the map) in the different professional fields, because it requires a sustained professional judgment in what Dreyfus and Dreyfus^6^ have called situational understanding (the territory).

Secondly, and as a consequence of this loss of confidence in the way it is explained and how professional expertise develops, there has been a great deal of dissatisfaction and there has been a lot of criticism of the training nurses receive. It is questioned that universities dominated by “monodisciplinary” cultures and formal knowledge production requirements are able to provide quality vocational training based on the knowledge actually used by professional nurses and the skills they effectively put into play for the development of all their functions^1^.

In order for this training to be effectively developed, university research is required to focus on the practice of care, a kind of phenomenological “return to reality”^2^. It is about studying the knowledge of professional nurses in their real contexts of elaboration and use: their work and the tasks they develop, their knowledge in practice^5^. This knowledge is inseparable from “professional activities”. They are studies elaborated and incorporated during the professional practice of care and only has sense in relation to it. The professional practice of nursing and the knowledge that sustains it, and that is produced in it, are not separate instances, but co-belong to a particular practice, co-evolve with it and transform it. Thinking about professional knowledge without articulating them with the practical situations that give them meaning is a mistake.

There is enough evidence to affirm that the practical situations faced by professional nurses are fraught with uncertainty and ambiguity, which can only be reduced by analyzing the situation and its context^1^ ^,^ ^3^ ^,^ ^7^. This analysis, however, is carried out both on the context of the situation and on the expectations, motivations, and interests of the participants in it, resulting in a “frame of reference” that contextualizes and delimits the possibilities of actions to be carried out and facilitates the understanding of problems. What is important to emphasize is that neither the original analysis nor the resulting framework is products of academic knowledge or the derivation and application of principles and technical rules assimilated by the professional nurse during him/her initial training^2^. In conclusion, it is claimed that the multiple pieces of knowledge and skills that support nursing professional practices are considered a source of the first order for the design and development of university programs for the training of nurses.
